# From Suspected COVID-19 to Anti-synthetase Syndrome: A Diagnostic Challenge in the Pandemic Era

**DOI:** 10.7759/cureus.52733

**Published:** 2024-01-22

**Authors:** Sérgio Gomes Ferreira, Luís Fernandes, Sara Santos, Sofia Ferreira, Mónica Teixeira

**Affiliations:** 1 Internal Medicine, Centro Hospitalar de Entre o Douro e Vouga, Santa Maria da Feira, PRT

**Keywords:** interstitial lung disease, immunomediated, covid-19, pneumonia, anti-synthetase

## Abstract

Anti-synthetase syndrome (ASS), a rare immunomediated disease, is characterized by multiple signs and symptoms. Not all patients develop the entire clinical spectrum of the syndrome, as it often varies depending on the involved antibodies. In this case report, a 53-year-old non-smoking woman had complaints of fatigue and dyspnea on exertion for five weeks. The outpatient study revealed creatine kinase (CK) 351U/L, ANAs+, anti-SSa+, normal echocardiogram, and a chest X-ray suggesting imaging suspicion of SARS-CoV-2 pneumonia. Referred to the emergency department, she was hospitalized for bilateral interstitial pneumonia without respiratory failure. Three SARS-CoV-2 polymerase chain reaction tests were negative. She underwent a five-day course of dexamethasone 6mg due to suspected coronavirus disease 2019 (COVID-19) sequelae with favorable progress. About a month later, she experienced fatigue, exertional intolerance, morning cough, and Raynaud's phenomenon episodes. Anti-SARS-CoV-2 antibodies were negative, and a follow-up chest CT showed bilateral organizing pneumonia. Bronchofibroscopy and bronchoalveolar lavage with cytology suggestive of inflammatory appearance, predominantly CD8+ lymphocytes, were performed. Subsequently, positive results for anti-OJ antibodies were obtained. A diagnosis of ASS was established, and prednisolone was initiated at 60mg/day with a tapering regimen, resulting in clinical and radiological improvement. Additional therapy with azathioprine was proposed. This case is presented due to highly suggestive COVID-19 imaging changes, emphasizing the importance of a high suspicion of ASS, despite nearly exclusive pulmonary involvement, with only one isolated elevated CK value and no musculoskeletal complaints. It is also noteworthy for the association with anti-OJ antibodies, rarely identified, often presenting interstitial lung disease as an isolated manifestation.

## Introduction

Anti-synthetase syndrome (ASS) is a rare autoimmune disease with a heterogeneous combination of clinical manifestations, including interstitial lung disease (ILD), myositis, polyarthritis, "mechanic's hands," and Raynaud's phenomenon [1]. This syndrome is associated with antibodies targeting aminoacyl-tRNA synthetases, triggering an autoimmune response leading to diverse tissue injuries [2]. The clinical expression of ASS is highly variable, predominantly influenced by the specific types of antibodies involved, however it distinguishes itself from other inflammatory myopathies by its significant lung involvement and its associated increased morbidity and mortality in these patients [3,4]. Additionally, some patients may not develop the full clinical spectrum of the syndrome, making diagnosis challenging and often delayed. Among the antibodies linked to ASS, anti-OJ is a rare and distinctive specificity, frequently associated with isolated ILD [5]. ILD, characterized by inflammatory changes in the alveolar spaces and pulmonary interstitium, is one of the most common and debilitating manifestations of ASS [6]. Its clinical presentation often mimics other pulmonary conditions, including sequelae of viral respiratory infections such as severe acute respiratory syndrome coronavirus 2 (SARS-CoV-2) [7]. Imaging findings of ASS based on high-resolution computed tomography (HRCT) are not specific and can include bilateral areas of ground glass, consolidation, and reticulation, with traction bronchiectasis pointing to the presence of underlying fibrosis. The corresponding patterns are non-specific interstitial pneumonia (NSIP), organizing pneumonia (OP), and mixed NSIP/OP, while a usual interstitial pneumonia pattern (typical or probable) has only rarely been described [8].

## Case presentation

**Figure 1:**
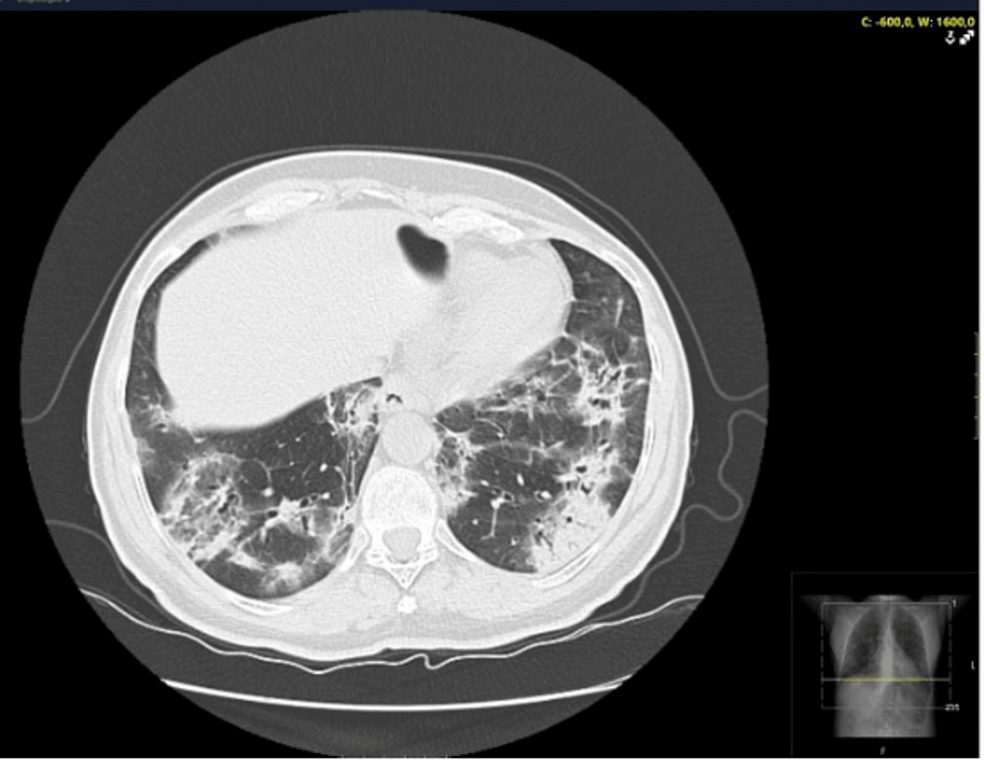
First chest CT scan at the time of the presentation in the Emergency Department.

**Figure 2:**
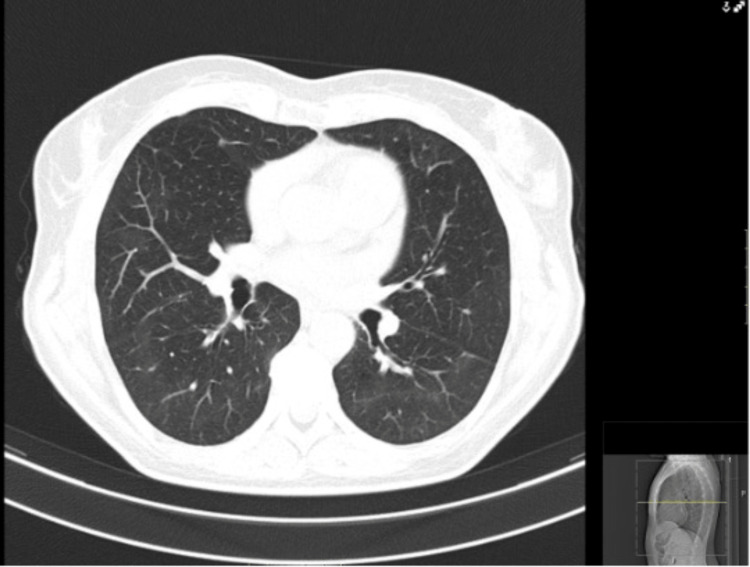
Follow-up chest CT scan, demonstrating a significant pulmonary improvement.

A 53-year-old non-smoking woman with no significant past medical, occupational and exposure history, nor chronic regular medication presented at the emergency department with complaints of fatigue and dyspnea upon minimal exertion with progressive worsening over a five-week period, during the coronavirus disease 2019 (COVID-19) pandemic season. Initially interpreted as anxiety, with no relevant findings regarding physical examination, laboratory results and chest X-ray, she was discharged with no drug prescription. An outpatient reevaluation raised suspicion of SARS-CoV-2 infection leading to another emergency department admission. Her vital signs were stable, with normal blood pressure and cardiac frequency, with oxygen saturation (SpO2) of 94% in breathing air. Physical examination had no significant findings, specifically regarding pulmonary auscultation. Lab test results showed no elevation of inflammatory markers (normal white blood cells and C-reactive protein (CRP)). A CT scan was performed, with findings of bilateral pulmonary infiltrates, suggestive of COVID-19 infection (Figure 1) with two SARS-CoV-2 as well as one multiplex virus test - by polymerase chain reaction (PCR)- all being negative for SARS-CoV-2, influenza A and B and respiratory syncytial virus. With a CORAD score of 4 (clinical and chest CT scan with high suspicion of COVID-19 infection) the patient was hospitalized for bilateral interstitial pneumonia without respiratory failure, she received a five-day course of dexamethasone, showing favorable progress and was discharged with an Internal Medicine reevaluation scheduled. The investigational study during outpatient evaluation revealed elevated creatine kinase (CK) of 351 U/L, positive antinuclear antibodies (ANAs), and anti-SSa antibodies. Pulmonary function tests were also done, with normal results. Approximately one month later, she experienced fatigue, exercise intolerance, morning cough, and Raynaud's phenomenon episodes. Despite negative anti-SARS-CoV-2 antibodies, a follow-up chest CT indicated bilateral organizing pneumonia. Bronchofibroscopy and bronchoalveolar lavage with cytology were performed, with negative bacterial or mycobacterial cultures, with findings suggestive of inflammatory changes, predominantly with CD8+ lymphocytes. Subsequent positive anti-OJ antibodies confirmed the diagnosis of ASS. Treatment with prednisolone at 60mg/day, followed by a tapering schedule, resulted in clinical and radiological improvement (Figure 2). Additional therapeutic consideration with azathioprine was proposed. 

## Discussion

This case prompts a nuanced discussion regarding the intricate diagnostic challenges posed by ASS, especially when presenting with imaging findings reminiscent of COVID-19 sequelae. The distinctive feature of predominantly pulmonary involvement, with only an isolated elevation of CK and the absence of musculoskeletal symptoms, underscores the diverse and often elusive nature of ASS. The association with the anti-OJ antibody in this case further accentuates the complexity of ASS. This antibody, infrequently identified, has been commonly linked to isolated ILD [9]. The clinical spectrum of ASS is broad, and the presence of specific antibodies, such as anti-OJ, can offer valuable diagnostic insights [2]. The correlation between imaging findings suggestive of COVID-19 and the ultimate diagnosis of ASS highlights the importance of maintaining a high index of suspicion for autoimmune conditions, even when initial clinical and radiological presentations mimic more common infectious etiologies [10]. The delayed recurrence of symptoms, along with the positive anti-OJ antibodies, emphasized the need for ongoing vigilance and a comprehensive diagnostic workup in the face of evolving clinical scenarios. Furthermore, the isolated elevation of CK without overt musculoskeletal complaints challenges traditional diagnostic paradigms associated with ASS. This case reinforces the necessity for a holistic clinical evaluation and consideration of less typical presentations to ensure timely and accurate diagnosis, facilitating the initiation of appropriate therapeutic interventions [11]. The intricacies of this case underscore the imperative for heightened awareness among clinicians, emphasizing the need for a multidimensional diagnostic approach in the context of complex autoimmune diseases like ASS [12]. As our understanding of these conditions evolves, so too must our diagnostic acumen to optimize patient outcomes through tailored therapeutic strategies.

## Conclusions

This case underscores the importance of considering ASS in patients with complex pulmonary manifestations, even in the absence of typical musculoskeletal symptoms. The diagnostic workup was even more challenging with imagiologic findings suggestive of SARS-CoV-2 infection during the COVID-19 pandemic. The detection of the rare anti-OJ antibody may be crucial for an accurate diagnosis and therapeutic management with corticosteroids and immunosuppressants proved effective in this case, highlighting the necessity of a multidisciplinary therapeutic approach to optimize clinical outcomes in patients with ASS.
